# Sustained nutrition impact of a multisectoral intervention program two years after completion

**DOI:** 10.1111/mcn.13103

**Published:** 2020-11-03

**Authors:** Anastasia Marshak, Helen Young, Anne Radday, Elena N. Naumova

**Affiliations:** ^1^ Feinstein International Center Tufts University Boston Massachusetts USA; ^2^ Friedman School of Nutrition Science and Policy Tufts University Boston Massachusetts USA

**Keywords:** international child health nutrition, low‐income countries, malnutrition, monitoring and evaluation, nutritional interventions, research methodology

## Abstract

Progress on the nutrition Sustainable Development Goals has been slow. More attention is needed on the ‘sustainable’ part, focused on impact lasting beyond programme implementation. To determine sustained impact of a multisectoral nutrition intervention that provided water, sanitation, hygiene, livelihood, health and nutrition support (2013–2015) in eastern Chad, we utilize longitudinal household data collected 2 years (2017) after the intervention ended. Between 2013 and 2015, children (6–59 months) in the multisectoral intervention were less likely to be severely wasted, underweight and had a higher weight‐for‐height *z*‐score (WHZ) compared with the control. To measure sustained programme impact, we use data on six nutrition indicators from 517 children between 2015 and 2017. We ran three models: a generalized linear model on cross‐sectional child cohorts; a mixed‐effects model on household panel data; and a mixed‐effects model on child panel data. For children who were born during the programme, we saw significant improvement in underweight, weight for age *z*‐scores (WAZs) and height‐for‐age *z*‐scores (HAZs). Boys 6–23 months born after the end of the programme, on the other hand, were significantly more likely to be underweight or wasted and had lower WHZ and WAZ compared with boys born during the programme and girls born during and after the programme. Corresponding to the literature from sub‐Saharan Africa, boys appear to be more vulnerable to malnutrition, which might be why they are more sensitive to programme cessation. Future monitoring, evaluations and research need to consider impact sustainability and that it might not be homogeneous across age and gender.

**Key messages**

- There is a limited body of evidence on sustained programme impact on child nutrition outcomes.
- Two years after a multisectoral intervention ended, children who were born into the programme showed improvement in height‐for‐age *z*‐score (HAZ), weight for age *z*‐score (WAZ) and underweight.
- Boys born after the cessation of the multisectoral intervention had significantly higher prevalence of wasting and underweight and a lower WHZ and WAZ compared with boys born into the intervention and girls of all age groups.
- Future evaluations need to build in long‐term monitoring after the programme is completed to understand whether gains achieved at the end of the programme are sustainable. Such evaluations should allow for sex and age disaggregation.

## 1 INTRODUCTION

The Sustainable Development Goals (SDGs) aim to reduce wasting to 5%, reduce stunting by 40% by 2025 and end all forms of malnutrition by 2030 (United Nations [UN], 2015). However, the global prevalence of wasting is declining slowly, with just 37 (19%) out of 194 countries on track to meet the 2025 target (Development Initiatives, 2018). In some regions, such as West Africa, stunting has actually increased from 2000 to 2015 (UNICEF, 2016) despite ongoing investment in development and humanitarian programming. Programmes and evaluations need to increase their focus on the ‘sustainability’ part of the SDGs if we are to meet the nutrition targets. In this paper, we analyse the sustained impact of a programme by asking do programme achievements, specifically in child nutrition, remain beyond the duration of the intervention itself and without continued input of external resources? We explore the sustained impact on the child level, for children born into the intervention, and at the household level to capture whether programme benefits remain for children born into intervention households, but after the completion of the programme. Through a literature review, we found only a handful of studies that evaluate the postprogramme impact on nutrition outcomes of a multisectoral intervention.

In 2012, Olney et al. conducted a literature review to identify programmes and platforms that deliver multiple micronutrient interventions using ‘sustainability’ as one of their seven criteria for effectiveness. This search turned up only one case study evaluating sustainability in agriculture (Olney, Rawat, & Ruel, 2012), but it looked at sustained use of gardens, not sustained impact on nutrition outcomes (Iannotti, Cunningham, & Ruel, 2009). A 1‐year postprogramme evaluation of a package of agricultural interventions with behaviour change communication strategies found evidence of a significant protective effect of the programme on preventing wasting among children born into treated households during the programme period (Dillon, Bliznashka, & Olney, 2020). Another evaluation of 8‐months postprogramme effects of a multisectoral intervention found a sustained impact on household food security, maternal dietary diversity and younger sibling's complementary feeding practices (Leroy, Olney, Bliznashka, & Ruel, 2020), but did not conduct an evaluation of nutrition outcomes. Finally, a 2015 evaluation of 12 multisectoral Food for Peace programmes across four countries—Bolivia, Honduras, India and Kenya—explored programme impact 2 to 3 years after exit (Rogers & Coates, 2015). Of the eight projects that used underweight as an outcome, four projects sustained the improvements in underweight, two projects made further improvement and two projects saw deterioration in the postprogramme impact. Overall, the evaluation found that the level of impact seen at the end of the programme did not predict how sustainable the impact would be in 2to 3 years. More so, according to Rogers and Coates (2015), the current practice of studying impact exclusively at the end of a programme jeopardizes investment in longer term sustainability. The limited existing studies on postprogramme impact sustainability of multisectoral interventions on nutrition outcomes underscore a serious gap in the literature.

Between 2013 and 2015, Concern Worldwide implemented the Community Resilience to Acute Malnutrition (CRAM) programme in Sila, Chad, with the goal of preventing acute malnutrition in children aged 6–59 months. CRAM integrated three programme sectors: water, sanitation and hygiene (WASH); health and nutrition; and livelihoods. Using a randomized control study design on 1,261 children spread across the control and treatment arm, an impact was observed on key household inputs (increased use of boreholes and latrines) and outputs (increase use and knowledge of hygiene behaviours across the water chain and hand washing) and nutrition outcomes (Marshak, Young, Bontrager, & Boyd, 2017; Marshak, Young, & Radday, 2016). Longitudinal panel analysis from 2013 to 2015 showed that the odds of a child being severely wasted were 76% lower (confidence interval [CI] [0.59, 0.86], *p* = 0.001), the odds of a child being underweight were 33% lower (CI [0.15, 0.48], *p* = 0.012) and weight‐for‐height *z*‐score (WHZ) was 0.18 standard deviations higher (CI [0.09, 0.28], *p* = 0.022) in the treatment group compared with the control (Marshak, Young, Radday, & Naumova, 2020). The impact evaluation and programme implementation were completed in 2015.

In 2017, researchers returned to the original treatment households and repeated the same survey to understand whether the initial impact was sustained or deteriorated with the cessation of programming in 2015. Given that the intervention was targeted on the village or household level, rather than child level, did not depend on the provision of food to improve nutrition outcomes and provided not only physical inputs but also knowledge and practice (Concern, 2011; Concern, 2017), we would expect for the nutrition programme impact to be sustained beyond the life of the intervention. We test this hypothesis by using the 2015 and 2017 postprogramme data from the original CRAM treatment villages. This paper presents findings on whether impact on nutrition outcomes was sustained 2 years after the completion of the multisectoral programme for both children born into the CRAM programme and children born after the end of CRAM but into the original treatment households.

## 2 METHODOLOGY

### 2.1 Setting and population

The Sila Region is in eastern Chad and is a home to a mix of livelihoods, including pastoralism, agro‐pastoralism and farming (Kratli, Sougnabe, Staro, & Young, 2018; Marshak et al., 2017). Among the sampled communities, most households depend on a combination of livestock rearing and rain‐fed cultivation. The area is extremely remote (950 km from the capital) and experiences one short rainy season from approximately May to August (Herrmann & Mohr, 2011; Marshak et al., 2016). The Sila Region historically has levels of acute malnutrition above the humanitarian emergency threshold of 15% (Young & Marshak, 2017).

### 2.2 Sample

The sample for the sustained programme impact study discussed in this paper comes from the original CRAM impact evaluation design (Marshak et al., 2020). The CRAM study sampling frame was a 2010 household list with household wealth rankings compiled by Concern Worldwide in the Goz Beida area of Sila as part of their blanket feeding and general food distribution. Seventy villages from the 2010 sampling frame had at least 20 households in the bottom wealth groups and hence were selected for the impact evaluation. The CRAM treatment was randomly assigned to each village, and approximately 20 households from the three bottom wealth groups were randomly selected in each community for the data collection. One treatment village refused to participate in the data collection (but still received the treatment), resulting in a total of 34 treatment villages. The CRAM sample included weight and height data on 1,261 children, 613 of which were in the treatment villages. The final sample allowed for a detection of a minimum difference of 7% in wasting between the control and treatment group with a power of 0.80, significance level of 0.05 and an interclass correlation of 0.06.

After the successful completion of the impact evaluation in 2015, no additional support was provided by Concern Worldwide to the former treatment villages. To measure the sustainability of the impact of the CRAM programme, another data collection was carried out in the former treatment villages in 2017. For this paper, we specifically focus on households in the former treatment villages from 2015 to 2017. In 2017, 7% of households were lost to attrition from the 2015 sample. No new households were added to the data collection in subsequent years. Therefore, across both 2015 and 2017, we have (depending on the nutrition outcome) data from approximately 1,000 children for the full child analysis, 680 children for the household panel and 200 children for the child panel (Table 1). We conducted ad‐hoc power calculations using a power of 0.80, alpha of 0.05 and an interclass correlation of 0.06 to determine the minimum detectable difference between 2015 and 2017 across our six nutrition indicators (Table S1) and three types of analysis. Each analysis approach is associated with an increase in the detectable minimum effect size (Table S2). For example, for wasting, the minimum detectable differences between 2015 and 2017 increase from 8% to 12% for the full child data analysis versus the child panel analysis.

**TABLE 1:** Sample size by model, age group and gender among children from Community Resilience to Acute Malnutrition (CRAM) communities in Sila, Chad, in 2015 and 2017

			Model 1: All child data			Model 2: Household panel			Model 3: Child panel		
			WHZ	HAZ	WAZ	WHZ	HAZ	WAZ	WHZ	HAZ	WAZ
Study total	2015		512	514	517	339	328	341	100	103	102
	2017		544	557	548	341	346	342	122	123	123
	Total		1,056	1,071	1,065	680	674	683	222	226	225
By age group	2015	6–23 months	132	134	134	129	130	131	56	59	57
		24–49 months	380	380	383	287	287	289	44	44	45
	2017	6–23 months	175	178	177	171	174	173	3	3	3
		24–49 months	369	379	371	231	295	289	119	120	120
	Total	6–23 months	307	312	311	300	304	304	59	62	60
		24–49 months	749	759	754	575	582	578	163	164	165
By gender	2015	Boys	240	239	241	193	193	194	44	44	44
		Girls	272	275	276	221	222	224	56	59	58
	2017	Boys	243	248	245	201	205	203	54	54	54
		Girls	301	309	303	238	244	238	68	69	69
	Total	Boys	483	487	486	394	398	397	98	98	98
		Girls	573	584	579	459	466	462	124	128	127

Abbreviations: HAZ, height‐for‐age *z*‐score; WAZ, weight for age *z*‐score; WHZ, weight‐for‐height *z*‐score.

### 2.3 Data collection

For the 2015 and 2017 data collection, enumerators participated in a 2.5‐week training on the survey tool, with 1 week allocated specifically to anthropometry data collection using the Standardized Monitoring and Assessment of Relief and Transitions (SMART) training guidance (UNICEF, 2017). Data were collected over 5 weeks in November and December 2015 and 2017. The enumerators conducted one‐on‐one interviews using a standardized questionnaire on a tablet, collecting information on household demographics, wealth, food security, decision making, access and utilization of health services, and child health (Marshak et al., 2016). Women served as the respondent for all points of data collection given the polygamous family structure and the women's role as primary caretaker of children. The enumerators used Salter scales and height boards to collect weight and height data from all children in the households between the ages of 6 and 59 months. Weight and height data measurements were collected three times to help increase accuracy and precision. Oral consent was required to participate in the study, and all instruments were approved by the Tufts University Institutional Review Board (IRB). Following IRB procedures, all data was de‐identified. A separate key linking the individual household identification with the respondent's name was kept securely on the primary researcher's server.

### 2.4 Data analysis

Prior to the analysis, we cleaned and examined all the household and child data using Stata/SE 13.1. After transforming the anthropometric data into *z*‐scores, we removed values with a *z*‐score greater than negative or positive 5 for WHZ and *z*‐scores greater than negative or positive 6 for height‐for‐age (HAZ) and weight for age (WAZ); thus, the sample size differs slightly by the indicator (Table 1).

To determine sustained impact, we ran three different types of analysis, each with its own strengths and weaknesses (discussed further below). Therefore, all three approaches together are required to validate and improve our understanding of whether the programme had a sustained impact on nutrition outcomes. All analysis was run on six outcome variables (Table S1). We used both the continuous and binary versions of each of our variable (i.e., wasting vs. WHZ) due to possible misclassification bias involved in interpretation of binary outcomes. The cut‐off for wasting is −2 standard deviations of WHZ; thus, children just above that would not be classified as wasted. Throughout the report, we use 95% CIs to show the level of uncertainty, but this would not capture the possible misclassification error, and hence, we report across both our binary and continuous outcomes. Given some inconsistencies in the oedema variable, we did not use it as part of our definition of wasting.

For Model 1, we took advantage of all the available data from 2015 to 2017 as if it was two cross‐sectional surveys and treated each child observation as independent across time. Initial analysis across time (2015 vs. 2017) and between different age groups (6–23 months vs. 24–59 months) and by gender across time was done using ordinary least squares (for continuous outcomes) and logit (for binary outcomes) regression on time. We controlled for sample design (village clustering) and applied population weights to adjust for the varying village population but equal sample size. Next, we replicated the model, but this time controlling for child gender and age (in months). Given that about 20% of children in this model include a 2015 and 2017 measurement, we also ran a sensitivity analysis on just the nonrepeated data to validate our approach (Table S3).

Model 2 uses the original design of the project, which followed the same households across time, to carry out a panel analysis on one child in the household. We ran a mixed‐effects regression on both the worst‐off and best‐off child in the household in regard to their nutrition outcomes (i.e., child with the lowest WHZ, wasted child in the household, etc.). In this model, we control for child gender, age (in months), the total number of children between the ages of 6–59 months in the household, and included household and village fixed effects. We then repeated the analysis, but separately for children 6–23 months versus 24–59 months. To further illustrate how boys born after the CRAM programme ended experienced a different trend in nutrition outcomes compared with boys born into the CRAM programme and girls of all age groups, we ran an additional model only for children 6–23 months with an interaction term between boys and the year 2017. Finally, we wanted to confirm that the deterioration in nutrition indicators observed for boys 6–23 months was universal rather than differential. To do so, we replicated the regression on children 6–23 months with the interaction terms between time and being a boy, but this time, controlling for household size, whether the household head was female, age of the household head, food insecurity (measured using the Months of Adequate Household Food Provisioning indicator, Bilinsky & Swindale, 2010), livestock per capita (measured using the tropical livestock units/per capita variable, Harvest Choice, 2015), number of assets owned and distance to a health centre (in minutes).

For Model 3, we identified children that were measured in both 2015 and 2017 to carry out child panel analysis. Because individual child codes were not collected, we used the following criteria to identify the same children across the two time periods:

1. Child had to be in the same household over time.
2. Child had to be the same gender over time.
3. Child had to be between 18 and 30 months older in 2017 compared with 2015. The 6‐month margin of error was used because capturing the true age of a child is extremely difficult in settings like eastern Chad due to lack of birth certificates. Instead, enumerators had to use seasonal and holiday calendars to estimate the age of a child resulting in a likely wide margin of error.

We then ran a mixed‐effects regression for the panel child sample, also adopted for both continuous and binary outcomes. We controlled for age (in months) and gender as well as household and village level fixed effects. For the child‐level panel analysis, we were only able to identify 21% of all children as the same in 2015 and 2017.

The following equations were used for Model 1 (all child data), Model 2 (household panel: one observation per household) and Model 3 (child panel) for binary and continuous outcomes:

**Table:** 

		Outcome	Equation
Model 1: All child data	Equation (1)	*Y*_*i*_	*β*_0_+*β*_1_*t*_*i*_+*β*_2_*g*_*i*_+*β*_3_*a*_*i*_+*β*_4_*g*_*i*_ × *t*_*i*_+*β*_5_*n*_*j*_+*ε*_*iv*_
	Equation (2)	$\text{logit}\left(P_{i}\right)=\ln\left(\frac{P_{i}}{1-P_{i}}\right)$	
Model 2: Household panel data	Equation (3)	*Y*_*ivt*_	*β*_0_+*β*_1_*t*_*i*_+*β*_2_*g*_*iv*_+*β*_3_*a*_*ivt*_+*β*_4_*g*_*iv*_ × *t*_*i*_+*β*_5_*n*_*ivt*_+*α*_*i*_+*ε*_*ivt*_
	Equation (4)	$\text{logit}\left(P_{\mathrm{ivt}}\right)=\ln\left(\frac{P_{\mathrm{ivt}}}{1-P_{\mathrm{ivt}}}\right)$	
Model 3: Child panel data	Equation (5)	*Y*_*ijvt*_	*β*_0_+*β*_1_*t*_*ijv*_+*β*_2_*g*_*ijv*_+*β*_3_*a*_*ijvt*_+*β*_4_*g*_*ijv*_ × *t*_*i*_+*β*_5_*n*_*jvt*_+*β*_6_***X***_*jvt*_+*α*_*i*_+*ε*_*ijvt*_
	Equation (6)	$\text{logit}\left(P_{\text{ijvt}}\right)=\ln\left(\frac{P_{\text{ijvt}}}{1-P_{\text{ijvt}}}\right)$	

where

*Y*_*ijt*_ is the continuous outcome variable for *i*‐child from *j*‐household in *v*‐village at *t*‐time (*t* = 0 for 2015 and *t* = 1 for 2017). In Equation (2), *j* equals *i* because we only have one observation per household (worst‐off or best‐off child).

*P*_*ijvt*_ is the probability of *i*‐child being wasted, stunted, or/and underweight from *j*‐household in *v*‐village at *t*‐time.

*t*_*ijv*_ represents time (2015 vs. 2017) for *i*‐child from *j*‐household in *v*‐village.

*g*_*ijv*_ represents the gender for *i*‐child from *j*‐household in *v*‐village (1 = female and 0 = male for all but the equation with the interaction term where 1 = male and 0 = female).

*a*_*ijv*_ represents the age of the child in months for *i*‐child from *j*‐household in *v*‐village at *t*‐time.

*g*_*ijv*_ × *t*_*ij*_ represents the interaction term between being a male and time for *i*‐child from *j*‐household in *v*‐village.

*n*_*jvt*_ represents the number of children between 6 and 59 months for *j*‐household in *v*‐village at *t*‐time.

***X***_*kijt*_ represents the vector of *k*‐control variables for *i*‐child from *j*‐household at *t*‐time.

*α*_*i*_ is the error term for *i*‐child.

*ε*_*ijt*_ is the error term for *i*‐child from *j*‐household in *v*‐village at *t*‐time.

The regression coefficients associated with time indicate that the changes in the outcome of interest are the main focus of this analysis. We show the average change over time along with 95% CIs.

Each analysis approach has its strengths and weaknesses, and hence, all three approaches together are required to validate and improve our understanding of whether the programme had a sustained impact on nutrition outcomes. Model 1 provides for the largest sample size and hence lowest detectable effect size. However, because Model 1 includes observations across the same individual child as well as multiple children in the same household, the analysis suffers from some lack of independence. Previous research indicates that child nutritional outcomes can vary widely within the same household (Marshak et al., 2020) likely related to variability in intra‐household resource allocation frequently characterized as ‘benign’ neglect (Hampshire, Panter‐Brick, Kilpatrick, & Casiday, 2009). Thus, in Model 2, we run a separate analysis on the best‐off and worst‐off child in the household according to Marshak et al. (2020), addressing the issue of independence and treating the household as the main unit of analysis, thus controlling for omitted household level characteristics, but not child level. The result is a further reduction in sample size and increase in detectable effect size. The most precise model—Model 3—addresses the issue of independence of observations as well as allows us to control for omitted child and household level characteristics, but results in a dramatic drop in sample size, increasing the detectable effect size. Thus, we rely on all three approaches to help validate CRAM sustained impact findings.

### 2.5 Ethical considerations

Oral consent was required to participate in the survey, and all instruments were approved by the Tufts Internal Review Board (IRB). The IRB protocol number is 1209010.

## 3 RESULTS

The prevalence of wasting across the complete child dataset (what was used for Model 1) decreased from 13.7% (CI [10%, 18%]) in 2015, at the completion of the CRAM intervention, to 11.5% (CI [6%, 17%]) in 2017, 2 years after the programme ended. While all six nutrition indicators showed an improvement across the two time periods, the change was not significant at *p* < 0.05 (Table 2). However, we observe a more nuanced impact when we disaggregate by age group (Table 2). For WHZ, HAZ, underweight and WAZ, children ages 24–59 months experienced a sustained and significant improvement 2 years after the programme ended (from 2015 to 2017). The largest difference is a drop from 30% (CI [24%, 37%]) of children 24–59 months being underweight in 2015 to only 23% (CI [18%, 28%]; *p* value = 0.03) in 2017. No significant improvement was observed for children 6–24 months by 2017. More so, although not significant, for all indicators except wasting which did not change over time, a deterioration is observed for the younger age group. Variation by gender is less apparent, with girls having a significant improvement in wasting (13% with CI [8%, 18%] to 7% with CI [2%, 11%]; *p* value = 0.01) and boys a significant improvement in HAZ (−1.8 with CI [−2.0, −1.6] *z*‐score to −1.5 with CI [−1.8, −1.2] *z*‐score; *p* value = 0.04).

**TABLE 2:** Summary statistics for nutrition indicators by gender, age group and time for all children with available anthropometry data in Community Resilience to Acute Malnutrition (CRAM) communities in Sila, Chad, in 2015 and 2017^a^

		Wasting (%)	WHZ	Stunting (%)	HAZ	Underweight (%)	WAZ
6–23 months	2015	15.1 [9.1, 21.0]	−0.67 [−0.99, −0.35]	35.6 [25.9, 45.4]	−1.46 [−1.79, −1.12]	24.0 [15.2, 32.8]	−1.23 [−1.47, −0.99]
	2017	15.0 [7.5, 22.5]	−0.92 [−1.15, −0.70]	42.0 [33.2, 50.9]	−1.70 [−1.99, −1.40]	33.8 [23.4, 44.2]	−1.55 [−1.82, −1.29]
24–59 months	2015	13.22 [8.9, 17.5]	−0.86* [−1.01, −0.70]	38.4 [31.8, 45.0]	−1.62* [−1.79, −1.45]	30.6* [24.0, 37.1]	−1.55** [−1.70, −1.41]
	2017	9.9 [3.9, 15.8]	−0.70 [−0.92, −0.48]	32.2 [24.0, 40.5]	−1.33 [−1.60, −1.07]	22.9 [17.9, 27.8]	−1.26 [−1.44, −1.08]
Boys 6–59 months	2015	15.1 [9.9, 20.4]	−0.78 [−1.07, −0.50]	43.4 [37.2, 49.4]	−1.83* [−2.02, −1.64]	34.5 [26.1, 42.8]	−1.56 [−1.73, −1.39]
	2017	18.0 [10.9, 25.2]	−0.97 [−1.17, −0.76]	37.8 [28.3, 47.3]	−1.53 [−1.85, −1.21]	34.6 [26.2, 42.9]	−1.50 [−1.73, −1.27]
Girls 6–59 months	2015	12.5* [7.5, 17.5]	−0.82 [−1.05, −0.59]	32.9 [25.5, 40.4]	−1.36 [−1.55, −1.16]	23.9 [19.8, 28.1]	−1.38 [−1.51, −1.24]
	2017	6.5 [2.0, 11.0]	−0.62 [−0.82, −0.42]	33.4 [27.7, 39.0]	−1.38 [−1.60, −1.17]	20.0 [15.1, 24.9]	−1.24 [−1.40, −1.07]
Boys 6–23 months	2015	18.4 [5.7, 31.0]	−0.51* [−1.11, 0.09]	32.8 [14.0, 51.6]	−1.44 [−2.06, −0.82]	21.2** [7.1, 35.3]	−1.02* [−1.65, −0.38]
	2017	28.9 [15.9, 42.0]	−1.34 [−1.69, −0.99]	49.5 [37.0, 62.0]	−1.84 [−2.32, −1.36]	53.1 [37.4, 68.8]	−1.92 [−2.31, −1.54]
Boys 24–59 months	2015	14.0 [9.3, 18.7]	−0.87 [−1.12, −0.63]	46.8 [38.5, 55.0]	−1.96*** [−2.33, −1.59]	38.9* [27.8, 50.0]	−1.74*** [−1.98, −1.50]
	2017	13.1 [5.6, 20.6]	−0.80 [−1.08, −0.51]	32.6 [19.2, 45.9]	−1.39 [−1.76, −1.02]	26.1 [14.7, 37.6]	−1.31 [−1.61, −1.01]
Girls 6–23 months	2015	12.8 [4.0, 21.6]	−0.79 [−1.17, −0.39]	37.5 [24.4, 50.6]	−1.46 [−1.83, −1.10]	26.0 [16.5, 35.4]	−1.38 [−1.67, −1.08]
	2017	4.6 [0.1–9.2]	−0.61 [−0.78, −0.45]	36.4 [26.4, 46.4]	−1.59 [−1.90, −1.28]	19.0 [10.5, 27.7]	−1.27 [−1.50, −1.04]
Girls 24–59 months	2015	12.4 [6.3–18.5]	−0.84 [−1.04, −0.64]	30.9 [21.4, 40.3]	−1.31 [−1.52, −1.11]	23.0 [17.4, 28.6]	−1.38 [−1.48, −1.27]
	2017	7.4 [1.9–12.9]	−0.63 [−0.87, −0.38]	32.0 [25.1, 38.9]	−1.29 [−1.56, −1.01]	20.4 [15.3, 25.5]	−1.22 [−1.41, −1.04]
Total	2015	13.7 [9.5, 18.0]	−0.80 [−0.98, −0.62]	37.6 [32.0, 43.3]	−1.57 [−1.71, −1.44]	28.7 [23.7, 33.8]	−1.46 [−1.59, −1.33]
	2017	11.5 [6.2, 16.7]	−0.77 [−0.95, −0.59]	35.3 [29.3, 41.2]	−1.44 [−1.65, −1.24]	26.3 [21.9, 30.6]	−1.35 [−1.50, −1.20]

Abbreviations: HAZ, height‐for‐age *z*‐score; WAZ, weight for age *z*‐score; WHZ, weight‐for‐height *z*‐score.

^a^ Mean with 95% confidence intervals in brackets controlling for community population size and village clustering.

* Significant difference between 2015 and 2017 at *α* < 0.05.

** Significant at *α* < 0.01.

*** Significant at *α* < 0.001.

When we controlled for child level characteristics and re‐ran Model 1, the results were the same, showing no significant change in the nutrition indicators over time (Table 3). However, Models 2 and 3 showed a much more optimistic picture. Model 2 showed that the nutritional status of the worst‐off child in the household had significantly improved HAZ, stunting, WAZ and underweight over time. Model 3 showed a significant improvement for WHZ, underweight and WAZ.

**TABLE 3:** Regression results by nutrition outcome indicator among children in Community Resilience to Acute Malnutrition (CRAM) communities in Sila, Chad, from 2015 to 2017^a^

Sample	Outcome	2017 vs. 2015	Age (months)	Female	Number of child <5	Constant	*N*
All child data (Model 1)	Wasting	−0.19 [−0.64, 0.24]	−0.01 [−0.02, 0.01]	−0.64** [−0.99, −0.30]		−1.18** [1.63, −0.73]	1,056
	WHZ	0.02 [−0.11, 0.17]	−0.00 [−0.01, 0.01]	0.15 [−0.03, 0.33]		−0.85** [−1.16, −0.53]	1,056
	Stunting	−0.10 [−0.46, 0.25]	−0.01** [−0.02, −0.00]	−0.33* [−0.61, −0.04]		0.15 [−0.30, 0.62]	1,071
	HAZ	0.12 [−0.12, 0.38]	0.00* [−0.01, 0.01]	0.31* [0.04, 0.57]		−2.03** [−2.36, −1.71]	1,071
	Underweight	−0.12 [−0.41, 0.17]	−0.01** [−0.02, −0.01]	−0.64** [−0.98, −0.29]		−0.13 [−0.49, 0.22]	1,065
	WAZ	0.10 [−0.05, 0.26]	0.00 [−0.1, 0.01]	0.22** [0.06, 0.38]		−1.62 [−1.91, −1.33]	1,056
Household panel data: worst‐off child (Model 2)	Wasting	−0.47* [−0.95, 0.01]	−0.01 [−0.02, 0.01]	−0.75** [−1.26, −0.23]	0.27 [−0.06, 0.61]	−1.84** [−2.87, −0.80]	680
	WHZ	0.13 [−0.01, 0.28]	−0.00 [−0.01, 0.01]	0.23** [0.08, 0.39]	−0.22* [−0.32, −0.11]	−0.73** [−1.‐6, 0.41]	680
	Stunting	−0.50** [−0.88, 0.12]	−0.02** [−0.03, −0.00]	−0.35* [−0.72, 0.02]	0.27* [0.02, 0.53]	0.42 [−0.32, 1.18]	674
	HAZ	0.37** [0.16, 0.58]	0.01* [0.00, 0.01]	0.23* [0.01, 0.44]	−0.23** [−0.37, −0.08]	−2.05** [−2.48, −1.62]	674
	Underweight	−0.10** [−0.17, −0.03]	−0.00* [−0.01, 0.01]	−0.15** [−0.22, −0.08]	0.06** [0.01, 0.11]	0.45** [0.31, 0.60]	683
	WAZ	0.22** [0.07, 0.37]	0.00 [−0.01, 0.01]	0.30** [0.14, 0.46]	−0.16** [−0.27, −0.06]	−1.70** [−2.01, −1.39]	683
Household panel data: best‐off child (Model 2)	Wasting	−0.07 [−0.67, 0.53]	−0.01 [−0.03, 0.01]	−0.54 [−1.13, 0.04]	−0.54* [−0.97, −0.11]	−0.94 [−2.12, 0.22]	680
	WHZ	0.06 [−0.09, 0.21]	0.00 [−0.01, 0.01]	0.02 [−0.13, 0.18]	0.15** [0.05, 0.26]	−0.95** [−1.29, −0.62]	680
	Stunting	−0.07 [−0.51, 0.36]	−0.01 [−0.03, −0.00]	−0.38 [−0.84, 0.07]	−0.76** [−1.13, −0.40]	0.94* [0.01, 1.88]	653
	HAZ	0.10 [−0.12, 0.33]	0.00 [−0.00, 0.00]	0.15 [−0.07, 0.37]	0.42** [0.27, 0.57]	−2.14** [−2.58, −1.69]	653
	Underweight	−0.28 [−0.71, 0.14]	−0.01 [−0.02, 0.01]	−0.35 [−0.77, 0.06]	−0.53** [−0.84, −0.22]	0.18 [−0.61, 0.99]	683
	WAZ	0.12 [−0.02, 0.27]	−0.01 [−0.01, 0.01]	0.06 [−0.08, 0.22]	0.23** [0.13, 0.33]	−1.59** [−1.89, −1.29]	683
Child panel data (Model 3)	Wasting	−1.33 [−1.29, 0.27]	0.01 [−0.05, 0.06]	−0.24 [−1.33, 0.85]		−1.93* [−2.68, −0.18]	222
	WHZ	0.59** [0.21, 0.96]	−0.01 [−0.02, 0.01]	0.06 [−0.24, 0.36]		−0.82** [−1.28, −0.41]	222
	Stunting	−0.87 [−2.18, 0.44]	−0.01 [−0.06, 0.03]	−0.11 [−1.17, 0.94]		−0.27 [−1.63, 1.09]	226
	HAZ	0.15 [−0.30, 0.61]	−0.00 [−0.02, 0.01]	0.06 [−0.33, 0.46]		−1.47** [−1.97, −0.98]	226
	Underweight	−1.75* [−3.41, −0.10]	0.01 [−0.05, 0.06]	−0.10 [−1.34, 1.12]		−1.15 [−2.84, 0.53]	225
	WAZ	0.42* [0.06, 0.79]	−0.01 [−0.02, 0.01]	0.11 [−0.19, 0.43]		−1.38** [−1.77, −0.99]	225

Abbreviations: HAZ, height‐for‐age *z*‐score; WAZ, weight for age *z*‐score; WHZ, weight‐for‐height *z*‐score.

^a^ Regression coefficient with 95% confidence intervals in brackets controlling for population size and village clustering.

*** Significant at *p* value < 0.001.

** Significant at *p* value < 0.01.

* Significant at *p* value < 0.05.

Across all three models, girls tended to have better outcomes compared with boys. The age of the child was also prominent with older children performing better on most indicators across all three models. Using both Models 1 and 2, children aged 6–23 months (Table S4) had no significant change in nutrition outcomes over time. For children 24–59 months (Table 4), on the other hand, there was a significant improvement across multiple nutrition indicators. We observed a significant improvement in WHZ, HAZ, underweight and WAZ in Model 1 and a significant improvement in stunting, HAZ, underweight and WAZ across both the best‐off and worst‐off child in the household in Model 2.

**TABLE 4:** Regression results by nutrition outcome indicator for children 24–59 months in Community Resilience to Acute Malnutrition (CRAM) communities in Sila, Chad, from 2015 to 2017^a^

Sample	Outcome	Year	Age (months)	Female	Number of child <5	Constant	*n*
All child data (Model 1)	Wasting	−0.31 [−0.89, 0.24]	−0.01 [0.04, 0.02]	−0.35 [−0.78, 0.07]		−1.22 [−2.57, 0.12]	749
	WHZ	0.14* [0.00, 0.29]	−0.01 [−0.01, 0.00]	0.10 [−0.05, 0.26]		−0.83** [−1.28, −0.38]	749
	Stunting	−0.30 [−0.76, 0.14]	−0.04** [−0.06, −0.03]	−0.37 [−0.78, −0.02]		1.60** [0.94, 2.27]	759
	HAZ	0.29* [0.05, 0.54]	0.02 [0.01, 0.03]	0.37* [0.01, 0.73]		−2.82** [−3.34, −2.31]	759
	Underweight	−0.41* [−0.76, −0.06]	−0.03** [−0.05, −0.02]	−0.56* [−1.11, −0.02]		0.97* [0.23, 1.71]	754
	WAZ	0.28** [0.13, 0.44]	0.01** [0.00, 0.01]	0.22 [−0.01, 0.46]		−2.04** [−2.39, −1.69]	754
Household panel data: worst‐off child (Model 2)	Wasting	−0.59* [−1.12, −0.06]	0.01 [−0.01, 0.03]	−0.28 [−0.79, 0.22]	0.09 [−0.25, 0.44]	−2.21** [−3.56, −0.86]	575
	WHZ	0.13 [−0.03, 0.30]	−0.01 [−0.01, 0.00]	0.19* [0.01, 0.37]	−0.08 [−0.20, 0.03]	−0.66 [−1.1‐, −0.21]	575
	Stunting	−0.69** [−1.10, −0.29]	−0.05** [−0.07, −0.02]	−0.20 [−0.58, 0.17]	0.01 [−0.27, 0.24]	1.89** [0.85, 2.94]	582
	HAZ	0.52** [0.28, 0.76]	0.02** [0.01, 0.03]	0.18 [−0.05, 0.43]	0.00 [−0.16, 0.16]	−3.03** [−3.63, −2.43]	582
	Underweight	−0.96** [−1.44, −0.48]	−0.04** [−0.07, −0.02]	−0.62** [−1.07, −0.16]	0.18 [−0.11, 0.48]	1.38* [−0.11, 0.48]	578
	WAZ	0.37** [0.21, 0.53]	0.01** [0.00, 0.01]	0.15 [−0.01, 0.32]	−0.05 [−0.16, 0.06]	−2.14** [−2.56, −1.73]	578
Household panel data: best‐off child (Model 2)	Wasting	−0.30 [−0.91, 0.31]	−0.00 [−0.03, 0.02]	−0.19 [−0.79, 0.39]	−0.32 [−0.74, 0.09]	−1.66* [−3.24, −0.09]	575
	WHZ	0.04 [−0.11, 0.20]	−0.00 [−0.01, 0.01]	0.03 [−0.14, 0.20]	0.13* [0.01, 0.25]	−0.93** [−1.39, −0.47]	575
	Stunting	−0.46* [−0.86, 0.06]	−0.03** [−0.05, −0.01]	−0.56* [−0.95, −0.16]	−0.39** [−0.67, −0.12]	1.96** [0.95, 2.97]	582
	HAZ	0.29* [0.05, 0.53]	0.01 [−0.00, 0.02]	0.31* [0.08, 0.55]	0.29** [0.13, 0.45]	−2.61 [−3.22, −2.00]	582
	Underweight	−0.66** [−1.12, −0.20]	−0.02* [−0.04, −0.00]	−0.49* [−0.92, −0.06]	−0.39* [−0.71, −0.07]	0.98 [−0.09, 2.06]	578
	WAZ	0.22** [0.06, 0.37]	−0.00 [−0.01, 0.00]	0.15 [−0.00, 0.30]	0.21** [0.10, 0.32]	−1.74** [−2.14, −1.34]	578

Abbreviations: HAZ, height‐for‐age *z*‐score; WAZ, weight for age *z*‐score; WHZ, weight‐for‐height *z*‐score.

^**a**^ Regression coefficient with 95% confidence intervals in parentheses controlling for population size and village clustering.

*** Significant at *p* value < 0.001.

** Significant at *p* value < 0.01.

* Significant at *p* value < 0.05.

**FIGURE 1:**
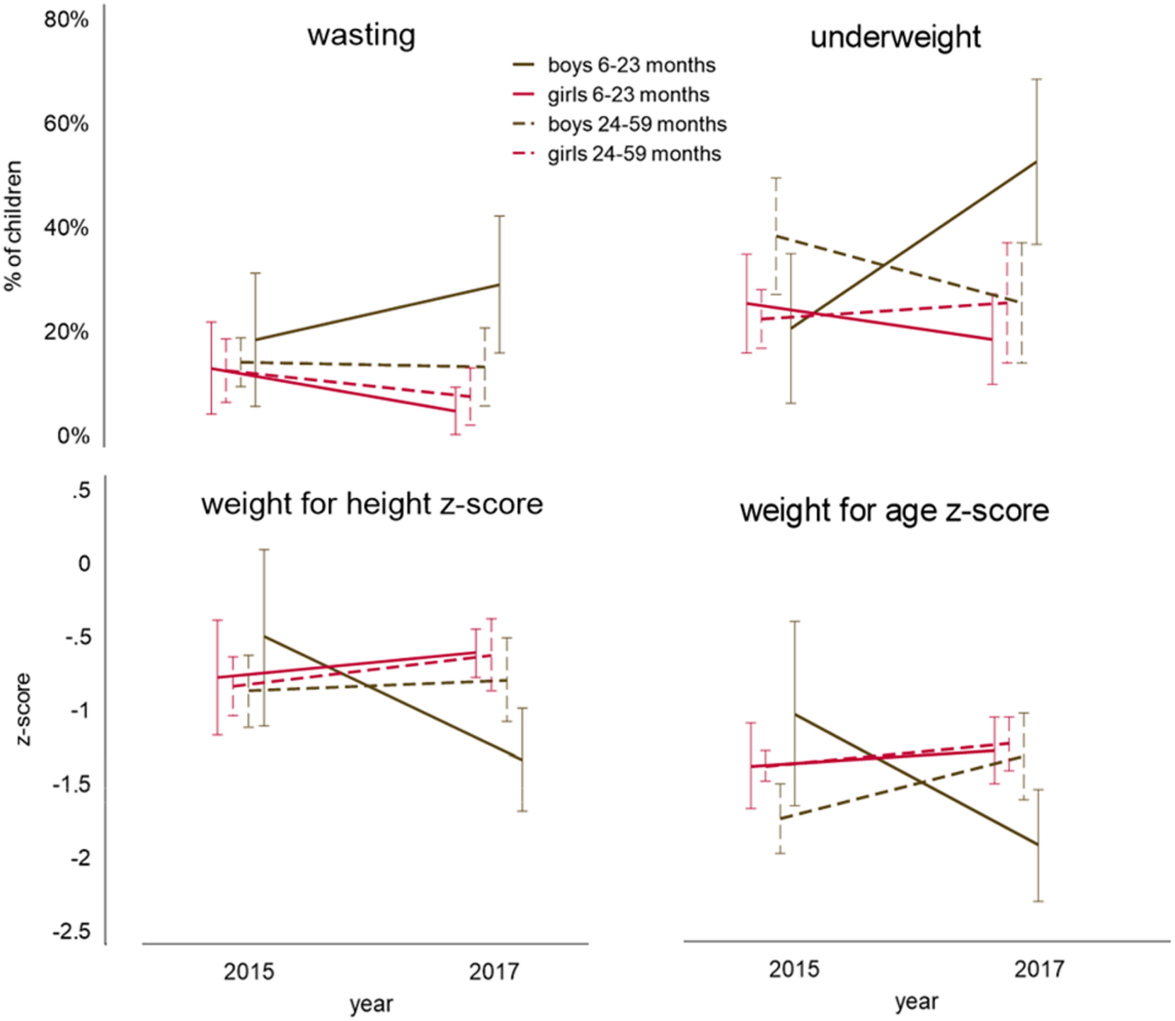
Change over time in wasting (weight‐for‐height *z*‐score [WHZ] <−2), WHZ, underweight (weight for age *z*‐score [WAZ] <−2) and WAZ by age and gender for all children with available anthropometry data in Community Resilience to Acute Malnutrition (CRAM) communities from 2015 to 2017 with 95% confidence intervals

Next, we looked at how much gender matters within the different age groups. As Figure 1 shows (using all the child data) that all gender age groups, except boys 6–23 months, perform similarly across time or show a slight improvement. On the other hand, boys 6–23 months show a deterioration in wasting, WHZ, underweight and WAZ. The findings are confirmed when running Models 1 and 2 only on children ages 6–23 months and including an interaction term between boys and time. For wasting, WHZ, underweight and WAZ, the interaction term is significant in Model 1; underweight remained significant for the worst‐off and best‐off child in Model 2 (Table S5).

The addition of household variables capturing household size, gender of household head, food insecurity, livestock, assets and distance to a health centre had no impact on the significance of boys under the age of 24 months in relation to wasting, WHZ, underweight and WAZ using Models 1 and 2 (Table S6). Thus, we can see that irrespective of a host of household characteristics, boys ages 6–23 months showed a significant deterioration in their nutritional status 2 years after the completion of the CRAM programme.

## 4 DISCUSSION

We find that programme impact appears to be sustainable, but *only* for children who were able to directly benefit from the programme during its implementation in line with findings from Dillon et al. (2020). Or put another way, individual child level impact was sustained, whereas household level impact (extending to children born after the CRAM programme) was not sustained. Specifically, the study found that the programme improved child nutritional indicators 2 years after the programme ended. However, the impact was not sustained for boys born after the programme ended. Boys aged 6–23 months in 2017 had significantly higher prevalence of wasting and underweight and a lower WHZ and WAZ compared with their age and gender group in 2015. The difference remained significant even when controlling for a host of household characteristics lending credence to the hypothesis that this might be a more generalizable finding.

The observed significant deterioration for boys 6–23 months is in direct contrast to the significant improvements in HAZ, underweight and WAZ for all children 24 months and older in 2017 (Models 1 and 2); significant improvement in stunting for the best‐off child in the household (Model 2); and significant improvement in stunting and wasting for the worst‐off child in the household (Model 2). The post‐2015 impact for children born during CRAM on HAZ and stunting (depending on the model) indicates that CRAM might have also had an impact on chronic malnutrition. Boys born after the end of CRAM, on the other hand, had their nutrition status deteriorate. Therefore, although we can say that the programme had an impact on all children who benefitted directly from it, it did not have an impact on household practices that would have sustained the improved nutritional status for boys who did not benefit directly from the programme.

This study adds to the growing literature identifying boys as more vulnerable or generally sensitive when it comes to nutrition outcomes. Across all three of our models, girls tended to have better nutrition outcomes compared with boys, which is consistent with the CRAM impact evaluation (Marshak et al., 2020). Numerous studies have reported similar findings in regard to stunting (Svedberg, 1990; Wamani, Astrom, Peterson, Tumwine, & Tylleskar, 2007), underweight, wasting and under‐five mortality (Svedberg, 1990) in the Sahel and in the Southeast Asia (Harding, Aguayo, & Webb, 2018). The gender disparity identifying boys as worse off is so consistent that the Wasting and Stunting Technical Group (WaST) highlighted it as one of seven key implications that need to be addressed: ‘in most contexts, boys are more wasted and stunted than girls. The reasons for this are unknown, but at a policy level this widespread finding indicates that common narratives around gender and heightened vulnerability of girls to malnutrition need to be revised’ (WaST, 2017). The gap in health outcomes across gender extends beyond childhood with men showing a lower life expectancy and the gap is only widening (Wang et al., 2012). The World Health Organization has called on global health institutions to address this gap in both policy and programming as part of the post‐2015 sustainable development agenda (Baker et al., 2014).

Considering that globally, girls are worse off on almost all metrics—school attendance, job security, food security, and so forth—their lower vulnerability when it comes to malnutrition is quite surprising, particularly in the Sahel, which is considered a highly patriarchal society that tends to favour males. Explanations for this phenomena range from favouritism for girls (Cronk, 1989) to more biological reasons that leave younger boys more vulnerable to mortality and morbidity (Elsmen, Pupp, & Hellström‐Westas, 2004). Research on environmental factors, such as pollution, for example, find a stronger negative effect on health outcomes among boys in early childhood and among women in adulthood (Clougherty, 2010). One particularly interesting explanation comes from Zinder, Niger. Qualitative field research on traditional knowledge and practice related to child care and feeding revealed that boys are weaned earlier because it is believed that breastmilk will make the child stupid (Howson, Harrison, & Law, 1996). While this practice might appear temporarily beneficial for girls, the motivation behind it is still related to preferential treatment for males. The authors are particularly interested in this explanation given similar findings from qualitative exploratory research with the CRAM population in 2019. Mothers reported that social practice dictates that boys should spend less time with the mother to grow into strong men and therefore are frequently weaned earlier (Marshak et al., 2020; in progress). Recent research out of India also finds that preferential treatment for boys can actually lead to worse nutrition outcomes, in this case, overweight and obesity (Naandi Foundation, 2014). It is possible that the differences in gender across the nutrition indicators in this study, and across sub‐Saharan Africa, reflect the cumulative effects of different beliefs and practices.

It is worth noting that there are several methodological limitations to this evaluation. First, we do not have a proper counterfactual for what happened to child nutritional status between 2015 and 2017 in communities that never received the multisectoral programme. Second, while we disaggregate the data by sex and age in the analysis, the study design was not originally set up to do this. Power calculations were set up for the whole sample, rather than for subgroups. The difference in sample size (fewer kids under 24 months vs. over 24 months), for example, could be a critical component of when we observe significant change over time. Therefore, we recommend that future studies make better considerations for gender and age stratification in the original study design and analysis (Mazurana, Benelli, Gupta, & Walker, 2011).

This study highlights the dangers of not including data collection points past programme implementation and organizational presence as part of initial programme evaluation design. Programmes might appear effective in reducing malnutrition in the short run when the reality is that malnutrition levels return to preprogramme conditions after cessation. Unless we explicitly incorporate the definition of sustainability as part of impact, as was written in the 1991 Development Assistance Committee Principles for Evaluation of Development Assistance (Organisation for Economic Co‐operation and Development [OECD], 1991), and use the appropriate study evaluation design, we might further backtrack in meeting the nutrition SDGs. Programmes and evaluations need to also consider that impact sustainability might not be homogenous by age and gender. Follow‐up research is required to understand why boys, even in patriarchal societies, are faring worse when it comes to nutritional status and possibly less likely to sustain benefits from programming with the aim of improving nutritional status.

## CONFLICTS OF INTEREST

The authors declare that they have no conflict of interest.

## CONTRIBUTIONS

AM designed the research. AM and AR conducted the research. AM performed the statistical analysis with support and discussion with HY and EN. AM wrote the article with HY and EN reviewing and editing the manuscript. All authors have read and approved the final manuscript.

## Supporting information


**Table S1:** Nutrition outcome variable definition and options
**Table S2**: Ad‐hoc power calculations: minimum detectable difference in nutrition outcomes between 2015 and 2017 by type of analysis in children in the CRAM program in Sila, Chad^†^
**Table S3**: Regression results by nutrition outcome indicator among children without repeated measurements across the two time periods in the CRAM communities in Sila, Chad from 2015 to 2017^†^
**Table S4**: Regression results by nutrition indicator for children 6–23 months from CRAM communities in Sila, Chad from 2015 to 2017^†^
**Table S5**: Regression results by nutrition outcome indicator for children 6–23 months in CRAM communities in Sila, Chad from 2015 to 2017 with interaction term between gender and time^†^
**Table S6**: Regression results for children 6–23 months in CRAM communities in Sila, Chad from 2015 to 2017 on wasting, WHZ, underweight, and WAZ including additional controls^†^
