# Supplementary material for: Sustained nutrition impact of a multisectoral intervention program two years after completion
Source: Matern Child Nutr. 2020 Nov 3;17(2):e13103. doi: 10.1111/mcn.13103 (PMC7988880; doi:10.1111/mcn.13103)
Supplement: Supplementary file 1 — Table S1: Nutrition outcome variable definition and options Table S2: Ad‐hoc power calculations: minimum detectable difference in nutrition outcomes between 2015 and 2017 by type of analysis in children in the CRAM program in Sila, Chad† Table S3: Regression results by nutrition outcome indicator among children without repeated measurements across the two time periods in the CRAM communities in Sila, Chad from 2015 to 2017† Table S4: Regression results by nutrition indicator for children 6–23 months from CRAM communities in Sila, Chad from 2015 to 2017† Table S5: Regression results by nutrition outcome indicator for children 6–23 months in CRAM communities in Sila, Chad from 2015 to 2017 with interaction term between gender and time† Table S6: Regression results for children 6–23 months in CRAM communities in Sila, Chad from 2015 to 2017 on wasting, WHZ, underweight, and WAZ including additional controls† [file MCN-17-e13103-s001.docx]

**Supplementary Tables & Figures**

**Supplementary figures 1 a-d):** Chromatograms for AFs & OTs in standards & human breastmilk


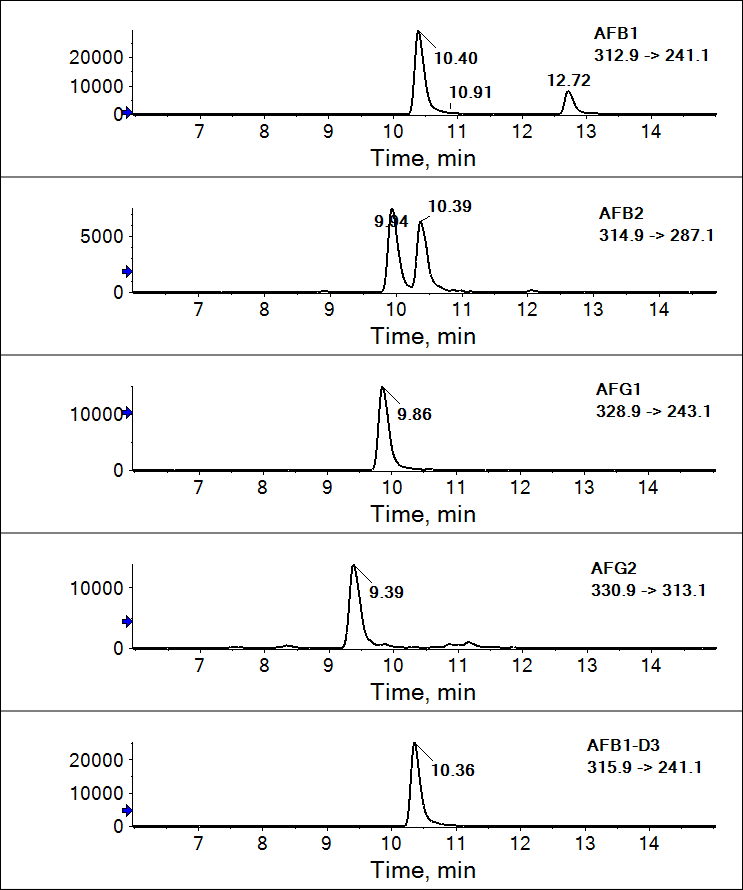

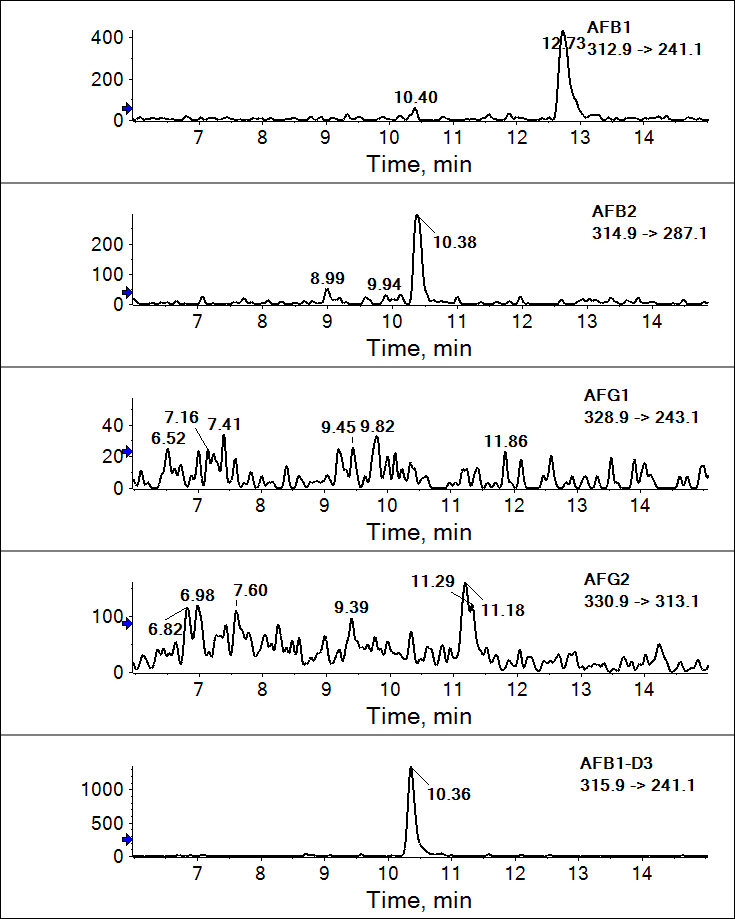


STDs

Human Milk

**INTENSITY**


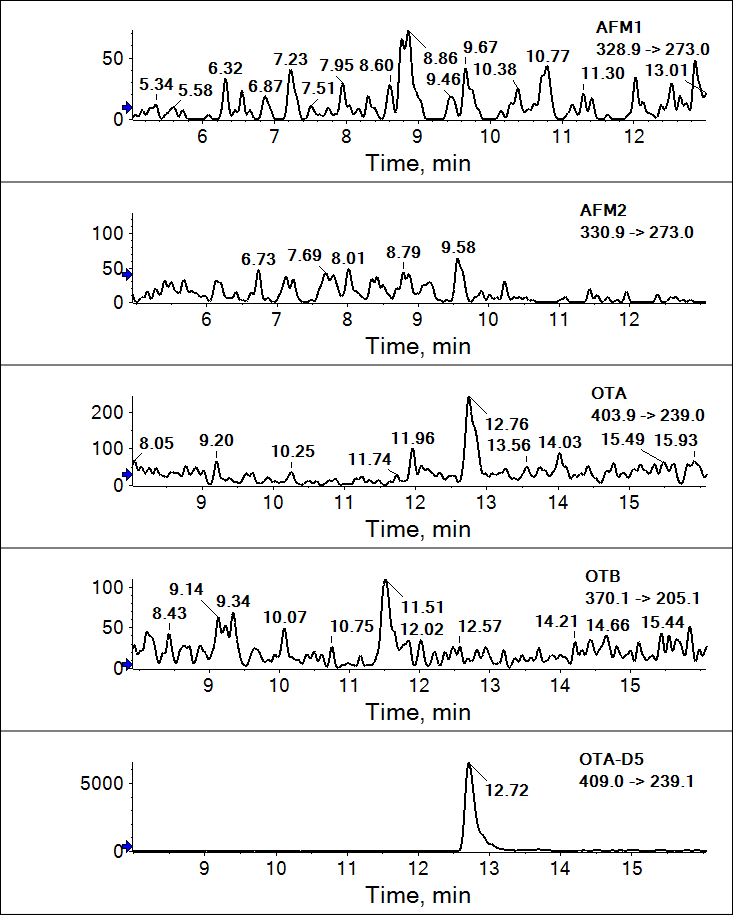


STDs

Human Milk


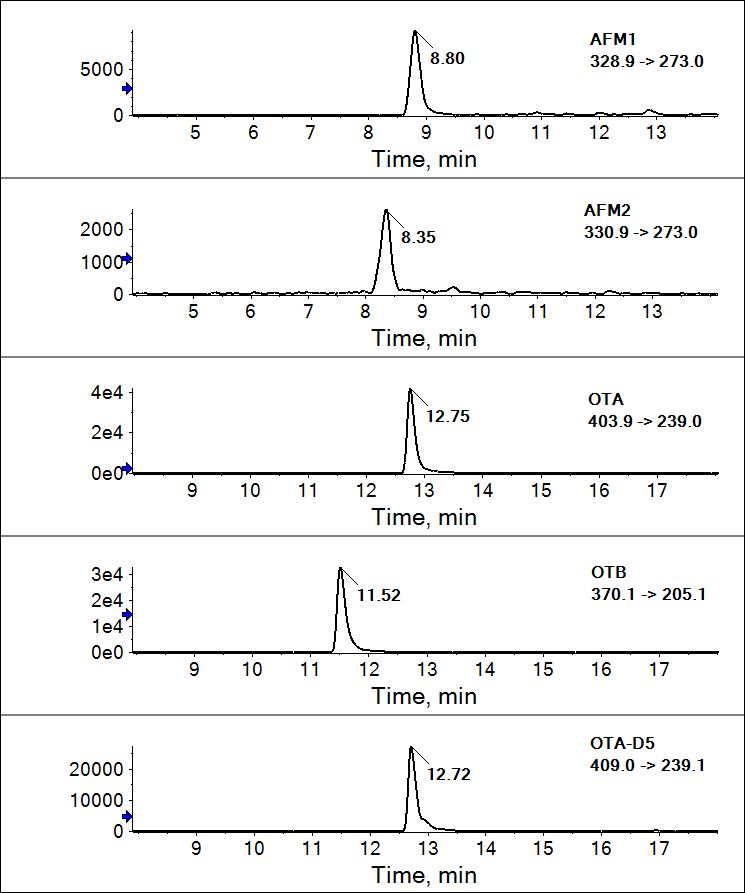


**INTENSITY**

**Supplementary figure 2):** Mycotoxin concentrations in animal milk samples (N=30)^a,b,c^


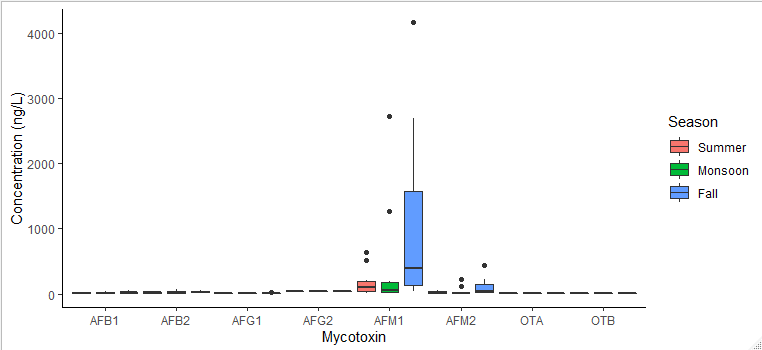


^a^Values for milk samples < LOD were replaced with 0.5 LOD value for each metabolite

^b^Fresh buffalo and commercial packaged cow milk samples tested

^c^No significant differences in AFM_1_, AFM_2_ levels by season

| **Supplementary Table 1a:** Concentrations of AFB_1_ in food items by season | | | | | | | | | | | | | | | |
| --- | --- | --- | --- | --- | --- | --- | --- | --- | --- | --- | --- | --- | --- | --- | --- |
| **Food Item** | **Regulatory Limit**  **(µg/kg)**^a^ |  | | **Summer** | | | **Monsoon** | | | | **Fall** | | | |  |
|  |  | **n** | **Median (IQR)** | | **Range** | **N > reg limit** | **n** | **Median (IQR)** | **Range** | **N > reg limit** | **n** | **Median (IQR)** | **Range** | **N > reg limit** | **p-value**^b^ |
| **Chilies** | 30 | 18 | 12.2 (21.0) | | (0, 302.3) | 2 | 16 | 16.0  (27.7) | (0, 61.3) | 4 | 18 | 16.83  (17.8) | (6.4, 48.4) | 5 | 0.75 |
| **Flour** | 15 | 13 | 1.38 (4.4) | | (0, 16.7) | 1 | 13 | 9.8  (60.8) | (1.6, 215.0) | 6 | 20 | 2.51  (2.6) | (0, 8.1) | 0 | *<0.01* |
| **Groundnuts** | 10 | 1 | 0 (0) | | - | 0 | 6 | 57.5  (146.3) | (5.7, 249.1) | 3 | 12 | 0  (0.6) | (0, 67.8) | 2 | *<0.05* |
| **Maize** | 15 | 4 | 234.72  (597.0) | | (0, 836.7) | 3 | 5 | 29.3  (24.2) | (0, 105.7) | 4 | 6 | 115.1  (239.8) | (3.7, 247.6) | 4 | 0.21 |
| **Pearl millet** | 15 | 8 | 5.82  (17.2) | | (0, 103.7) | 2 | 10 | 55.6  (135.2) | (1.2, 160.2) | 7 | 10 | 0 (0) | 0 | 0 | *<0.01* |
| **Rice** | 15 | 35 | 0  (2.8) | | (0, 16.0) | 1 | 24 | 15.9  (49.8) | (0, 195.6) | 13 | 22 | 0 (0) | (0, 10.4) | 0 | *<0.0001* |
| **Sorghum** | 15 | 7 | 0  (3.0) | | (0, 6.2) | 0 | 3 | 8.4  (63.5) | (0, 63.5) | 1 | 5 | 0 (0) | 0 | 0 | 0.077 |
| **Wheat** | 15 | 12 | 0  (2.6) | | (0, 34.9) | 1 | 12 | 71.5  (130.9) | (1.3, 196.0) | 7 | 13 | 1.83  (0.4) | (1.4, 2.7) | 0 | *<0.0001* |
| **Infant Formula** | - | 10 | 0 (0) | | (0, 1.3) | - |  |  |  |  |  |  |  |  |  |

^a^Regulatory limits set by the Food Safety and Standards Authority of India

^b^p-value for f-statistic (ANOVA) examining differences in [AFB_1_] by season

| **Supplementary Table 1b:** Concentrations of FB_1_ in food items by season | | | | | | | | | | | | | | | | | | |  |
| --- | --- | --- | --- | --- | --- | --- | --- | --- | --- | --- | --- | --- | --- | --- | --- | --- | --- | --- | --- |
| **Food Item** | **Regulatory Limit**  **(µg/kg)**^a^ |  | | **Summer** | | |  | | | **Monsoon** | | |  | | **Fall** | | | **p-value**^b^ |  |
|  |  | **n** | **Median (IQR)** | | **Range** | **N > reg limit** | | **n** | **Median (IQR)** | | **Range** | **N > reg limit** | **n** | **Median (IQR)** | | **Range** | **N > reg limit** |  | |
| **Maize** | 2000^c^ | 4 | 5.7 (35.2) | | (0, 61.4) | 0 | | 5 | 33.4 (34.3) | | (0, 54.6) | 0 | 6 | 0 (0) | | (0, 23.6) | 0 | 0.29 | |
| **Pearl Millet** | - | 7 | 8.1 (22.6) | | (0, 30.4) | 0 | | 6 | 12.8 (17.6) | | (0, 35.4) | 0 | 10 | 0 (0) | | 0 | 0 | *0.015* | |
| **Sorghum** | - | 4 | 20.3 (19.5) | | (1.9, 29.9) | 0 | | 1 | 33.2 (0) | | - | 0 | 5 | 0 (0) | | 0 | 0 | *0.0097* | |

^a^Regulatory limits set by the Food Safety and Standards Authority of India

^b^p-value for f-statistic (ANOVA) examining differences in [FB_1_] by season

^c^Regulatory limits set for raw maize grain by JECFA/FAO

| **Analyte** | **Parent ion (m/z)** | **Product ion (m/z)** | **Retention time (min)** | **De-clustering Potential (volts)** | **Collision Energy (CE)** | **CXP (volts)** |
| --- | --- | --- | --- | --- | --- | --- |
| AFB_1_ | 312.93 | 241.08 | 10.4 | 161 | 53 | 30 |
| AFB_2_ | 314.95 | 287.08 | 9.9 | 176 | 39 | 26 |
| AFG_1_ | 328.92 | 243.05 | 9.9 | 161 | 39 | 22 |
| AFG_2_ | 330.96 | 313.13 | 9.4 | 131 | 35 | 32 |
| AFM_1_ | 328.96 | 273.04 | 8.8 | 171 | 33 | 38 |
| AFM_2_ | 330.92 | 273.05 | 8.4 | 146 | 33 | 32 |
| AFB_1_-D_3_ | 315.95 | 241..09 | 10.3 | 186 | 33 | 28 |
| OTA | 403.94 | 239.02 | 12.7 | 61 | 21 | 20 |
| OTB | 370.06 | 205.06 | 11.5 | 76 | 27 | 22 |
| OTA-D_3_ | 409.02 | 239.05 | 12.7 | 16 | 33 | 32 |

**Supplementary Table 2:** Selected Reaction Monitoring Table

**Supplementary Table 3:** Performance characteristics of LC-MS/MS analytical method

| **Analyte** | **Calibration range** | **R^2^** | **LOD** | **LOQ** |  | **Recovery** | **CV** |
| --- | --- | --- | --- | --- | --- | --- | --- |
|  | **(pg/mL)** |  | **(pg/mL)** | |  | **(%)** | **(%)** |
| **AFB_1_** | 15.6-1000 | 0.9995 | 15.6 | 31.3 |  | 99.3 | 5.91 |
| **AFB_2_** | 15.6-1000 | 0.9995 | 15.6 | 31.3 |  | 96.0 | 3.73 |
| **AFG_1_** | 15.6-1000 | 0.9999 | 15.6 | 31.3 |  | 100.75 | 2.76 |
| **AFG_2_** | 78-5000 | 0.9998 | 78 | 156 |  | 101.67 | 0.67 |
| **AFM_1_** | 7.8-500 | 0.9998 | 7.8 | 15.6 |  | 99.49 | 3.80 |
| **AFM_2_** | 7.8-500 | 0.9999 | 7.8 | 15.6 |  | 98.72 | 3.90 |
| **OTA** | 15.6-1000 | 0.9963 | 15.6 | 31.3 |  | 100.51 | 3.87 |
| **OTB** | 7.8-500 | 0.9935 | 7.8 | 31.3 |  | 98.46 | 5.88 |

%CV = coefficient of variation

| **Supplementary Table 4:** Median intake from FFQs of commonly consumed food items | | | |
| --- | --- | --- | --- |
|  | | **Item Intake** | |
| **Item** | **% Consumed** | **Daily Frequency**  **Median (min, max)** | **Times per week**  **Median (min, max)** |
| ***Breads & Rotis*** | | | |
| Tandoori roti | 12 | 1 (1,2) | 1 (1,1) |
| Roti | 95 | 7 (6,7) | 2 (1,3) |
| Paratha | 29 | 2 (1,7) | 1 (1,2) |
| Stuffed paratha | 17 | 1 (1,3) | 1 (1,3) |
| Bajra roti | 7 | 1 (1,7) | 1 (1,2) |
| Poori | 20 | 1 (1,2) | 1 (1,1) |
| Buns | 22 | 3 (1,7) | 1 (1,2) |
| Burgers | 9 | 1 (1,2) | 1 (1,1) |
| ***Grains & Staples*** | | | |
| Rice | 50 | 2 (1,7) | 1 (1,3) |
| Pulao | 20 | 1 (1,6) | 1 (1,2) |
| Mutton biryani | 8 | 1 (1,2) | 1 (1,2) |
| Khichdi | 15 | 1 (1,2) | 1 (1,2) |
| Dalia | 12 | 2 (1,5) | 1 (1,3) |
| Chow-mein | 15 | 1 (1,3) | 1 (1,1) |
| Kheer | 15 | 1 (1,7) | 1 (1,3) |
| ***Dairy & Animal Products*** | | | |
| Tea | 86 | 7 (1,7) | 2 (1,6) |
| Plain milk | 62 | 7 (1,7) | 1 (1,3) |
| Lassi | 31 | 2 (1,7) | 1 (1,2) |
| Butter | 5 | 3 (1,7) | 1 (1,3) |
| Ghee | 53 | 7 (1,7) | 2 (1,4) |
| Eggs | 18 | 1 (1,2) | 1 (1,1) |
| Curd | 22 | 2 (1,7) | 1 (1,2) |
| Paneer | 13 | 1 (1,6) | 1 (1,2) |
| Raita | 8 | 1 (1,4) | 1 (1,1) |
| ***Fruits & Vegetables*** | | | |
| Banana | 20 | 2 (1,5) | 1 (1,2) |
| Apple | 25 | 1 (1,7) | 1 (1,1) |
| Grapes | 7 | 1 (1,4) | 1 (1,1) |
| Mango | 14 | 2 (1,4) | 1 (1,2) |
| Watermelon | 8 | 1 (1,4) | 1 (1,1) |
| Guava | 6 | 1 (1,3) | 1 (1,1) |
| Jackfruit | 6 | 1 (1,1) | 1 (1,2) |
| Pomegranate | 10 | 1 (1,4) | 1 (1,1) |
| Juice | 6 | 1 (1,2) | 1 (1,1) |
| Salad | 10 | 2 (1,7) | 1 (1,1) |
| Pickle | 12 | 1 (1,3) | 1 (1,1) |
| Potato | 95 | 4 (1,7) | 2 (1,3) |
| Carrot | 21 | 1 (1,4) | 2 (1,3) |
| Cabbage | 19 | 1 (1,3) | 1 (1,2) |
| Okra | 29 | 1 (1,4) | 1 (1,3) |
| Cauliflower | 43 | 1 (1,5) | 2 (1,3) |
| Bottle Gourd | 43 | 1 (1,5) | 1 (1,4) |
| Brinjal | 47 | 1 (1,2) | 1 (1,3) |
| Peas | 37 | 1 (1,5) | 2 (1,2) |
| Capsicum | 11 | 1 (1,2) | 2 (1,2) |
| Chilli chutney | 12 | 2 (1,7) | 1 (1,2) |
| Tomato Chutney | 25 | 1 (1,3) | 1 (1,2) |
| Kaddu | 15 | 1 (1,2) | 1 (1,2) |
| Kathal | 6 | 1 (1,1) | 1 (1,2) |
| Leafy vegetables | 29 | 1 (1,3) | 1 (1,3) |
| ***Legumes*** | | | |
| Arhar | 11 | 2 (1,7) | 1 (1,3) |
| Other dals | 60 | 2 (1,7) | 1 (1,3) |
| Chana | 28 | 1 (1,3) | 1 (1,3) |
| Kadhi | 33 | 1 (1,2) | 1 (1,3) |
| Gram flour | 14 | 1 (1,1) | 1 (1,2) |
| Urad | 7 | 1 (1,2) | 1 (1,2) |
| Beans | 15 | 1 (1,2) | 1 (1,2) |
| ***Meats*** | | | |
| Chicken curry | 27 | 1 (1,5) | 1 (1,3) |
| Mutton curry | 8 | 1.5 (1,4) | 2 (1,2) |
| Fish curry | 6 | 1 (1,3) | 1 (1,2) |
| ***Sweets & Snacks*** | | | |
| Sugar | 38 | 7 (1,7) | 1 (1,3) |
| Jaggery | 18 | 4.5 (1,7) | 1 (1,3) |
| Chips | 9 | 1 (1,4) | 1 (1,1) |
| Snacks | 41 | 1 (1,7) | 1 (1,1) |
| Nuts | 15 | 2 (1,7) | 1 (1,2) |
| Samosa | 13 | 1 (1,3) | 1 (1,1) |
| Salted biscuits | 9 | 2 (1,3) | 1 (1,1) |
| Sweet biscuits | 49 | 1 (1,7) | 1 (1,3) |
| Pakora | 6 | 1 (1,1) | 1 (1,2) |
| Jalebi | 13 | 1 (1,2) | 1 (1,2) |
| Barfi | 19 | 1 (1,3) | 1 (1,1) |
| Milk sweets | 9 | 1 (1,2) | 1 (1,4) |
| Halwa | 14 | 1 (1,4) | 1 (1,2) |
| Cola | 23 | 1 (1,3) | 1 (1,2) |
